# Heart Rate and Body Temperature Evolution in an Interval Program of Passive Heat Acclimation at High Temperatures (100 ± 2 °C) in a Sauna

**DOI:** 10.3390/ijerph20032082

**Published:** 2023-01-23

**Authors:** Jesús Siquier-Coll, Ignacio Bartolomé, Mario Pérez-Quintero, Víctor Toro-Román, Francisco J. Grijota, Marcos Maynar-Mariño

**Affiliations:** 1 SER Research Group, Center of Higher Education Alberta Giménez, Comillas Pontifical University, Costa de Saragossa 16, 07013 Palma Mallorca, Spain; 2 Department of Sport Science, Faculty of Education, Pontifical University of Salamanca, C/Henry Collet, 52-70, 37007 Salamanca, Spain; 3 Faculty of Sport Sciences, University of Extremadura, Avenida de la Universidad s/n, 10003 Caceres, Spain

**Keywords:** heat stress, heat exposure, heat acclimation, heart rate, core temperature, skin temperature

## Abstract

Heat exposure provokes stress on the human body. If it remains constant, it leads to adaptations such as heat acclimation. This study aims to observe the evolution of heart rate (HR), core temperature (Tcore), and skin temperature (Tskin) in an intervallic program of exposure to extreme heat. Twenty-nine healthy male volunteers were divided into a control group (CG; *n =* 14) and an experimental group (EG; *n* = 15). EG experienced nine sessions (S) of intervallic exposure to high temperatures (100 ± 2 °C), whereas CG was exposed to ambient temperatures (22 ± 2 °C). HR, Tskin, and Tcore were monitored in S1, 4, 5, 8, and 9. An important increase in HR occurred in the S4 compared to the rest (*p* < 0.05) in EG. A lower HR was discovered in S8 and S9 compared to S4 and in S9 in relation to S1 (*p* < 0.05) in EG. EG experiences a gradual decrease in Tcore and Tskin, which was detected throughout the assessments, although it was only significant in the S8 and S9 (*p* < 0.05). Interval exposure to heat at 100 ± 2 °C elicits stress on the human organism, fundamentally increasing Tcore, Tskin, and FC. This recurring stress in the full program caused a drop in the thermoregulatory response as an adaptation or acclimation to heat.

## 1. Introduction

Heat is an important stressor for the human body, favoring several physiological reactions [1]. Acute heat accelerates the peripheral blood flow to the skin and reinforces sweating for cooling [2]. Furthermore, heat stress triggers a rise in heart rate (HR), a reduction in stroke volume, and hypovolemia [3]. This process intensifies cardiac output. Thermal stress also deepens the ventilation of the respiratory system [4].

Elsewhere, heat exposure has been used for different purposes due to the benefits heat acclimation (HA) produces on the human body. HA originates adaptations by increasing plasma volume. In addition, it has been proven to aid endothelial function by lowering blood pressure [5]. This feature is activated by the development of cutaneous microvascular function via nitric oxide [6]. As it has been observed in athletes, HA could enhance aerobic performance by improving maximum oxygen consumption (VO_2max_) in both heat and cold [7], as it stimulates the hypoxia-inducible factor-1 (HIF-1) factor [8]. 

Currently, different protocols for HA have been established. They usually consist either of a combination of exercise in heat at 25–35 °C and at an intensity of 50–60% of VO_2max_ or a heat exposure between 40 and 50 °C after exercise [9]. Nevertheless, other alternative strategies for exposure to heat at extreme temperatures intend to replace exercise stress with heat stress. These protocols are based on a passive heat exposure in a Finnish sauna or heat chamber at 50–90 °C and 20–30% relative humidity (RH) [10]. Heat stress in the absence of exercise equally triggers stimulation of the HSP72 protein [11]. Iguchi et al. (2012) stated that repeated passive exposure to heat is associated with protein changes, leading to improved metabolic health as well as protection from stressors [12]. These upgrades are similar to those obtained from regular physical exercise [13]. Recent investigations reported changes in FC after repeated sauna applications [14,15]. Nevertheless, these studies were carried out in patients with cardiac diseases or combined with physical exercise. Thus, the effects of a high temperature program in healthy young people are still unclear.

Previous research has reported that passive HA at high temperatures (<70 °C; 20–30% RH) induces similar adaptations to active HA [16,17]. However, a small number of studies have analyzed the effects of exposure to higher temperatures on the human organism. In this study, the short- and long-term responses in this system are hypothesized to be alike to those resulting from physical exercise if the stress produced by heat works fundamentally in the cardiovascular system as physical exercise does. Therefore, the objective is to assess the evolution of core temperature (Tcore), skin temperature (Tskin), and HR during interval exposure to sweltering conditions in a sauna at 100 ± 2 °C.

## 2. Materials and Methods

### 2.1. Participants and Ethical Approvals

Twenty-nine euhydrated male students voluntarily engaged in this research. All of them signed a form, giving their consent to effectuate this study. The participants were randomly divided into the Control Group (CG; *n* = 14) and the Experimental Group (EG; *n* = 15). This investigation was approved by the bioethics committee of the University of Extremadura (Registration number 01/2017) under the ethical guidelines of the Helsinki Declaration of 1975, updated by the 2013 World Medical Assembly in Fortaleza (Brazil), for research involving humans. For the collection and treatment of the samples, a code was assigned to each participant to preserve the anonymity of the subjects. Table 1 contains the main characteristics of the volunteers.

### 2.2. Experimental Protocol

EG underwent 9 sessions (S) (3 Weeks) of heat exposure in sauna at 100 ± 2 °C and 20–30% RH, whereas CG performed the same protocol but in a laboratory at 22 °C (40–50% RH). These sessions were conducted from 9 A.M. to 14 P.M. and at the same time for each participant to control circadian rhythms on alternate days. Tcore, Tskin, and HR from every participant were measured in sessions 1 (S1; first S of week 1), 4 (S4; first S of week 2), 5 (S5; second S of week 2), 8 (S8; second S of week 3), and 9 (S9; third S of week 3).

### 2.3. Exposure Sessions

EG performed the heat exposure in a sauna (Harvia C105S Logix Combi Control; 3–15 W; Finland). These sessions comprise a passive interval exposure of 3 sets of 10 min at 100 ± 2 °C (20–30% RH) with an intra-set recovery of 5 min at laboratory temperature (22 °C, 40–50% RH). This protocol was similar to the previous one conducted by Siquier-Coll et al. (2019) [18]. CG experienced laboratory temperature (22 °C, 40–50% RH) simultaneously with the EG. Every volunteer remained in a seated upright position in each session and was allowed to drink ‘ad libidum’.

### 2.4. Heart Rate and Temperature Assessment

The internal temperature in the mucosa of the mouth and the external temperature of the foreheads were measured with an infrared thermometer (TAT 5000 “Exergen Temporal Scanner” (EXERGEN, Waterrown, USA)) at the beginning and end of each set. For the external assessment, the forehead was previously cleaned to eliminate sweat.

HR was recorded by heart rate monitors (Team system; Polar; Finland). When the data was recorded, the mean HR of the first, fifth, and last minute of each set was analyzed. In addition, the mean HR of the last minutes of recovery between each set was recorded.

### 2.5. Health Security Protocol 

This protocol was similar to the suggested one by Siquier-Coll et al. (2019) [18]. Preceding the experimental period, every participant was examined by a physician to avoid any cases of illness or contraindication for this study. At this point, they had to comply with the inclusion criteria: being a healthy male, not having taken any supplementation, medication or over-the-counter medication, drug or alcohol in the previous week or during the investigation, having a healthy lifestyle, not practicing more than 3 h of physical activity per week, and not following a specific training plan.

The health security protocol contains an evaluation of the cardiocirculatory system of each subject in resting conditions with an electrocardiography (Sanro BTL-08 SD ECG, SANRO; Madrid, Spain) and a tensiometer (visiomat; comfort 20/40; UEBE Medical GmbH; Bürgermeister-Kuhn-Strasse, Germany). Previous to the exposure sessions, a physician verified the basal electrocardiograms to avoid cases of breathing difficulties. In addition, a spirometer (Spirobank G; MIR; New Berlin, USA) was used to examine respiratory capacity. Two forced spirometry tests were conducted before the exercise tests. No diseases were reported during the whole study.

### 2.6. Body Composition

The anthropometric measurements were collected in the morning, under fasting conditions, and at the same time for each participant. Body height was evaluated with a wall stadiometer (Seca 220; Hamburg, Germany). Body weight, fat-free mass, and fat mass were estimated by electric bioimpedance with a body composition analyzer BF-350 (Tanita; Tokyo, Japan).

### 2.7. Statistical Evaluation

Statistical assessments were performed with SPSS 22.0 for Windows. The results are expressed as the mean and standard deviation. The Kolmogorov–Smirnov test was applied to scrutinize the distribution of the variables, and Levene’s test was implemented to verify their homogeneity. Non-parametric tests were employed to associate the results because not all parameters reached normality. The difference between normothermia and hyperthermia, the former and the latter before and after intervention, and pre-post difference data were determined with the Wilcoxon test for paired samples in both groups. A *p* ≤ 0.05 was considered statistically significant.

## 3. Results

The data referring to the Tcore and Tskin are displayed in Table 2.

Tcore is higher at the beginning of S1 and S9 compared to S8 (*p* < 0.05). The subsequent Tcore to set 2 in S9 is lower than in S4 (*p* < 0.05), whereas the prior one to set 3 in S3 is inferior to that in S1 (*p* < 0.05). A higher Tskin was discovered at the beginning of S1 with respect to S5 (*p* < 0.05), S8 (*p* < 0.01), and S9 (*p* < 0.05). Likewise, previously to set 2 in S9, Tskin was massively minor than in S1 (*p* < 0.05). There were noteworthy differences between Tskin before and after comparison to S4 (*p* < 0.05).

Concerning intra-sets comparisons, Tcore raises in sets 2 and 3 in relation to set 1 in S1, S4, S5, and S8. In S9, Tcore before set 2 (*p* < 0.05) and pre-and-post set 3 were warmer than set 1 (*p* < 0.01; *p* < 0.05).

There were pre-post variations in Tskin (*p* < 0.05) and Tcore (*p* < 0.05) in every set of each session except for the pre-post set 3 Tcore of S1.

In Table 2, no weighty differences were found in the temperatures of CG.

**Figure 1:**
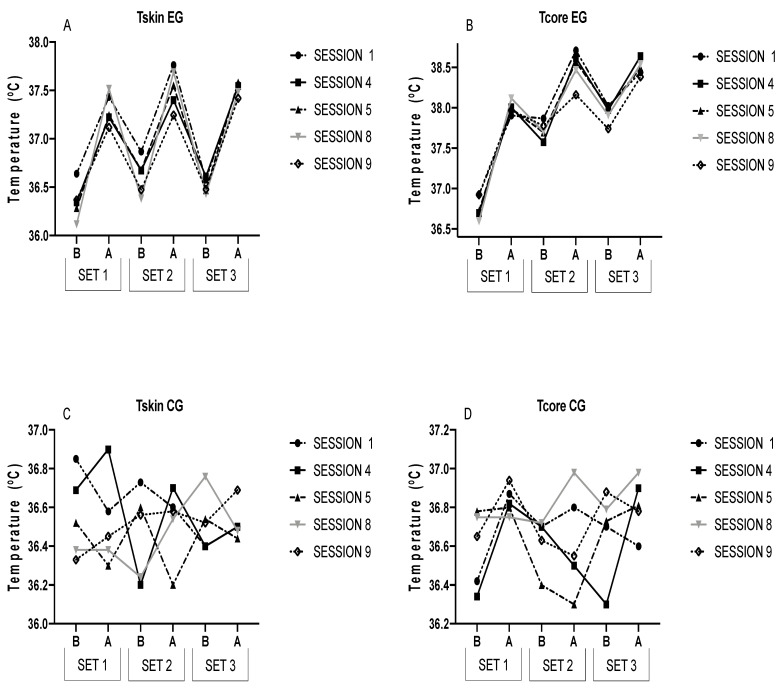
This figure describes Tcore and Tskin in each session in both groups. B = Before; A = After.

Figure 1 graphically presents the results of both groups for a better visualization.

The results of HR obtained from EG are expressed below in Table 3, and the CG results are presented in Table 3.

HR_R2_ and HR_3_ beat more rapidly than HR_R1_ in S1 (*p* < 0.05). HR_1_ from set 2 increased with respect to set 1 (*p* < 0.05). HR_R1_ of sets 2 and 3 accelerates in comparison with set 1 in S5 (*p* < 0.05). A rise in HR_10_ for set 3 is noticeable with respect to set 1 in S5 (*p* < 0.05). Besides, HR_1_ of set 3 suffered an increment in relation to set 1 (*p* < 0.05). In S8, HR_5_ of set 2 was quicker than in set 1 (*p* < 0.05), and HR_1_, HR_5_, and HR_10_ intensified in set 3 with respect to set 1 (*p* < 0.05). HR_10_ of sets 2 and 3 were higher in comparison with set 1 in S9 (*p* < 0.05).

HR_1_ and HR_10_ of set 1 in S4 were the most accelerated in the experiment (*p* < 0.05). HR_5_ of set 1 in S4 was swifter than in S1 and S9. 

Elsewhere, HR_R1_ of S4 was prompter than in S1, S5 and S9 (*p* < 0.05).

Concerning set 2, HR_1_ and HR_5_ of S4 were the most remarkable (*p* < 0.05). Similarly, HR_5_ of S4 surpasses S8 and S9 (*p* < 0.05). HR_10_ of set 2 in S5 and S9 was significantly lower than S4 (*p* < 0.05). 

No differences were reported in HR_R2_ between sessions.

Regarding set 3, a higher HR_1_ in S4 appears with respect to S8 (*p* < 0.05). There were differences in S4 in comparison to the other ones in HR_5_ of set 3 (*p* < 0.05). Likewise, HR_10_ of set 3 was lower in S1, S8, and S9 with respect to S4 (*p* < 0.05).

HR_R3_ in S4 exceeds S1, S8, and S9 (*p* < 0.05).

No considerable statistical differences were detected in Table 3.

**Figure 2:**
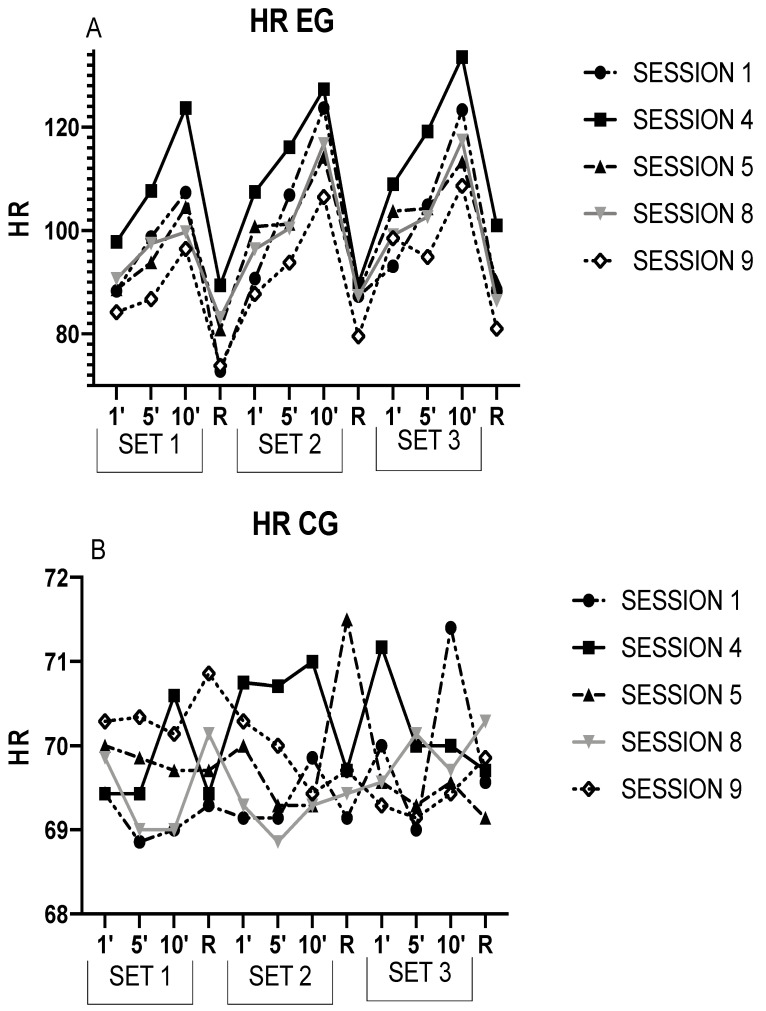
This figure shows the HR during each session in the EG (**A**) and the CG (**B**). R = Recovery. 1′ = minute 1; 5′ = minute 1; 10′ = minute 10.

Figure 2 graphically represents the results of the EG (A) and CG (B) for a better visualization.

## 4. Discussion

Passive HA protocols outstand due to the hotness participants are subjected to. Specifically, in a sauna, heat exposure should be above 70–80 °C [19]. Apparently, no evidence of a protocol where participants are exposed to 100 ± 2 °C has been documented yet, so the novelty of this research lies here.

The purpose of this investigation was to replace the stimulus of interval physical training with interval training where heat was the stressor and to discover whether it had a similar evolution or not. Therefore, successive changes had been detected in Tcore, Tskin, and HR in subjects during this stress-intensive interval heat program.

The first reflex in humans in warm environments is to quicken blood flow to the periphery to relieve the skin from heat [20]. The rise in temperature is linearly related to the acceleration in HR, increasing by 40% due to a hotter heart temperature, whereas 60% results from the activity of the autonomic nervous system [21]. Thus, the direct effects of ventricular contraction on nodal cardiac cells (sinoatrial and atrioventricular), the conduction speed, impulse propagation through the heart, and sympathetic and parasympathetic impacts on cardiac nodal cells are the determinants of the consequences of controlling and regulating HR and subsequent ventricular contraction during heat stress [22]. Elsewhere, the cardiac temperature rise could diminish phase IV of repolarization, increasing HR [22]. Another factor probably expediting HR is the circulation of catecholamines produced by heat stress, indicating an elevation in the adrenal medulla [23]. These possible mechanisms would increase HR in EG HR during heat exposure experiences.

The 1 °C rise in Tcore boosts the HR by 7–9 beats per minute (bpm) [21]. In this investigation, when Tcore grows ~1 °C, HR averagely increased ~10 bpm at 5 min and ~20 bpm at 10 min of exposure in S1 and S4 in the first set, being aggravated in sets 2 and 3. This phenomenon would indicate an accumulation of fatigue due to heat stress. This is equivalent to the data of interval training with an accumulation of exhaustion after periods of rest, so the hypothesis of cardiovascular stress by heat being similar to stress by exercise, with its limitations, could be demonstrated.

Leppaluoto et al. (1986) noted an acceleration in HR of around 20–30 bpm after 30 min of passive heat exposure at 80 °C in a sauna, with an augmentation in rectal temperature of 0.8–1 °C [24]. Similarly, another study observed an HR intensification until 140 ± 11 bpm after leaving subjects in a sauna at 89.9 ± 2.0 °C for 31 ± 5 min [25]. However, it was performed after training, so participants would accumulate fatigue. Recent research suggests a 15-bpm increment after a 30 min exposure to 73 ± 2 °C in a sauna with an elevation of tympanic temperature from 36.4 ± 0.5 to 38.4 ± 0.7 °C [26]. In this research, the HR recorded at the end of the third set in S4 was 133.54 ± 21.34 bpm, showing exhaustion due to heat interval exposure and growth in Tcore around 1 °C and Tskin of 2 °C. In this investigation, interval heat stress would act as interval training with accumulated fatigue in each set [27]. It could possibly be consistent because HR_R2_ and HR_R3_ beat more rapidly than HR_R1_ in S1 and S4, although no statistical changes were reflected in the Tcore.

Elsewhere, the acute responses in S4 were superior to the rest. This phenomenon could be due to the three acclimation sessions per week performed by the participants, and cardiovascular changes were reported to occur in the second week, where S4 would be the beginning of the second week [28]. Therefore, the increased reactions at the cardiac level could be explained by the attempt at an adaptive response to exposure to sweltering hotness. Curiously, it is in S5 when HR decreases with respect to S4 and S1, so the organism would start to adapt to extreme warmth. In both Figure 1 and Figure 2, as of S5, the HR, Tcore, and Tskin begin to decline, indicating an acclimation to heat.

The descent of HR in S5, S8, and S9 in relation to S4 could be multifactorial: a thermoregulatory adaption, for it also decreases Tcore and Tskin. Thus, thermoregulatory responses would be lower, eliciting a lessening in autonomic system stimulation. Besides, the cardiac temperature incrementation would be minor, so phase IV of repolarization would be intensified in S5, S8, and S9 than in S1 and S4. Another possible mechanism could be stimulation of the HIF-1 factor in EG [29]. HA could influence this factor, inducing an elevation of haemoglobin, haematocrit, and VO_2max_ [10]. These processes could facilitate the oxygen pulse, consequently decreasing HR. A recent study reported improvements in ventilation after HA, which may be another factor in HR reduction during the last sessions of heat exposure [16]. Likewise, greater blood perfusion to the skin favors nitric oxide-dependent arterial dilation in conditions of heat stress [6] and moderates arterial stiffness [30]. This could improve vascular function. These mechanisms could induce an enhancement in cardiac metabolic efficiency. Furthermore, a study suggests that sauna treatment at 60 °C for three weeks in patients with chronic heart failure decreased baseline HR, causing an increase in the left ventricular injection fraction [31]. Additionally, adaptation syndrome, whereby a certain stress factor in the environment leads to greater tolerance to this stressor [17,32], would also be the cause of the decrease in HR, Tcore, and TSkin.

Heat adaptions are suggested to arise with an augmentation of 1.5–2 °C in Tcore [4,16,17]. Nonetheless, an investigation reported that the aforementioned increase in Tcore is not necessary for there to be adaptations [33]. In this research, heat adaptions occurred with an increment of around 1 °C in Tcore and 2 °C in Tskin. 

Parallelly to these findings, Leppaluoto et al. (1986) observed a momentous HR drop in S6 and S7 with respect to the first one and in rectal temperature after the fourth one of heat exposure [24]. A recent review reported that 75–80% of HA adaptions develop between the fourth and seventh days of heat exposure [28]. In this study, these adaptions appeared in S5 and were higher in S9. Thus, EG may have acclimated to heat in S9. Nevertheless, CG did not suffer any change in HR, Tskin, or Tcore in normothermic exposure, inferring that the changes in EG were caused by repeated exposure to heat. Regarding Tcore, passive HA is evidenced to diminish it in recovery [34]. Figure 1 illustrates a gradual lessening in Tcore throughout the sessions in recovery, but only with significant differences between S1 and S9.

Recent research compared the effect of heat exposure for 25–30 min at 65–80 °C to a Tcore boost of 1.5–2 °C for 24 sessions, whereas the control group performed interval training. In conclusion, the group performing the hyperthermia sessions received superior ergospirometric improvements than the group performing interval training [16]. Another recent study with a similar methodology obtained the same results [17]. Hence, the initial hypothesis considered that heat stress would produce adaptations similar to physical exercise. Therefore, the current literature, along with the outcome of this study, suggests the possible veracity of this premise. Another recent study conducted with a similar protocol to this study, which found similar improvements with medium-intensity exercise in women [35].

In addition, this protocol of exposure to heat, performed at lower degrees Celsius (70–90 °C), could be implemented in populations with reduced mobility or with any heart problem preventing the individual from physical exercise. Vuori (1988) reported that the workload of the heart is minor with heat stress than with exercise, despite also accelerating HR [36]. During heat stress, blood pressure does not increase as it does with exercise [26], suggesting that controlled heat stress may be another option for health benefits, even in people with cardiac pathologies. 

Elsewhere, it could be observed that the behavior of temperature is similar to that of HR throughout HA. The main reason for the decrease in Tcore and Tskin is the rate of sweating. It has been documented that HA causes an increase in sweating rate [37]. A higher sweating rate results in better heat loss through the skin by evaporation. The increase in sweating rate occurs after 3–4 days [28]. This phenomenon is stimulated by the fact that HA produces a decrease in the sweating threshold [38]. Additionally, this increase in sweating is favored by increased vasodilatation, which promotes sweating. Thus, Figure 1 shows a noticeable decrease in the series from one session to the next. Another reason for the reduction of heat stress is the cardiovascular adaptations explained above, together with other hematological, sudomotor, and hematological adaptations [28]. Furthermore, it has been suggested that thermotolerance is another adaptation that allows cells to survive in environments with increased heat stress [39,40]. It is caused by the response of heat shock proteins (HSPs) [41], especially HSP72, which is sensitive to both exercise and heat [11]. HSP has been reported to be increased after heat acclimation. A study observed increases at rest after 15 days of HA [33]. Otherwise, it has been suggested that alterations in HSP90 may induce increased vasodilation [42,43], which would aid heat loss through sweating. These mechanisms would have helped induce lower Tcore and Tskin.

However, this study has limitations, such as the restricted quantity of samples. Further research should shed light on this protocol by involving a larger number of participants and diverse populations. Another limitation of this study was that the amount of water ingested by the participants “ad libidum” was not measured. This fact could have affected the thermoregulation of the participants.

## 5. Conclusions

Exposure to high warmth has a momentous stressful effect on the human organism, strongly increasing HR. Thus, this protocol could be an option to substitute physical exercise as a stressor and produce beneficial effects similar to this.

Interval exposure to hot temperatures produces a decrease in Tcore, Tskin, and FC after three weeks, corresponding to adaptations to heat.

## Figures and Tables

**Table 1:** Characteristics of the participants.

Parameters	EG (*n* = 15)	CG (*n* = 14)
Age (years)	22.34 ± 1.88	21.75 ± 1.71
Height (m)	171.86 ± 6.12	174.41 ± 4.67
Weight (Kg)	69.56 ± 6.41	70.82 ± 5,51
HRb (bpm)	79.43 ± 20.09	68.91 ± 11.48
Fat mass (kg)	11.96 ± 3.29	11.15 ± 2.47
Fat mass (%)	17.00 ± 3.55	15.63 ± 2.43
Fat-free mass (kg)	57.6 ± 4.08	59.66 ± 3.66
Fat-free mass (%)	83.00 ± 3.55	84.37 ± 2.43

EG = Experimental group; CG = control group; HRb = Baseline Heart Rate.

**Table 2:** Tcore and Tskin during S1, S4, S5, S8, and S9 of this study.

			EG				CG			
			Tcore	*p*-Value	Tskin	*p*-Value	Tcore	*p*-Value	Tskin	*p*-Value
S1	Set 1	Before	36.64 ± 0.63	0.001	36.92 ± 0.62	0.009	36.42 ± 0.56	0.085	36.85 ± 0.71	0.312
		After	37.43 ± 0.84		37.91 ± 0.58		36.87 ± 0.37		36.58 ± 0.35	
	Set 2	Before	36.87 ± 0.70 **	0.003	37.87 ± 0.64 *	0.013	36.70 ± 0.31	0.128	36.73 ± 0.28	0.159
		After	37.76 ± 1.19 **		38.71 ± 0.78		36.80 ± 0.44		36.60 ± 0.47	
	Set 3	Before	36.58 ± 0.65 **	0.074	38.02 ± 0.51	0.005	36.70 ± 0.37	0.327	36.40 ± 0.67	0.108
		After	37.56 ± 0.97 *		38.44 ± 0.79		36.60 ± 0.41		36.50 ± 0.34	
S4	Set 1	Before	36.34 ± 0.32	0.000	36.69 ± 0.68	0.000	36.34 ± 0.32	0.066	36.69 ± 0.68	0.096
		After	37.22 ± 0.58		38.01 ± 0.82		36.82 ± 0.67		36.90 ± 0.61	
	Set 2	Before	36.67 ± 0.53 **	0.001	37.57 ± 0.82 *	0.003	36.70 ± 0.54	0.098	36.20 ± 0.76	0.059
		After	37.40 ± 0.73 **		38.62 ± 0.61		36.50 ± 0.38		36.70 ± 0.67	
	Set 3	Before	36.61 ± 0.65 **†	0.003	37.97 ± 0.61 *	0.005	36.30 ± 0.49	0.254	36.40 ± 0.58	0.061
		After	37.54 ± 0.88 **		38.64 ± 0.79 *		36.90 ± 0.31		36.50 ± 0.28	
S5	Set 1	Before	36.28 ± 0.42	0.000	36.72 ± 0.57 ¥	0.000	36.78 ± 0.36	0.088	36.52 ± 0.28	0.073
		After	37.24 ± 0.52		37.99 ± 0.64		36.80 ± 0.21		36.30 ± 0.62	
	Set 2	Before	36.68 ± 0.51 **	0.002	37.69 ± 0.45 *	0.006	36.40 ± 0.65	0.346	36.60 ± 0.45	0.455
		After	37.55 ± 0.75 *		38.57 ± 0.89		36.30 ± 0.52		36.20 ± 0.74	
	Set 3	Before	36.47 ± 0.71 **†	0.022	38.01 ± 0.90 *	0.000	36.73 ± 0.35	0.061	36.54 ± 0.53	0.316
		After	37.58 ± 0.87 **		38.49 ± 0.68 *		36.81 ± 0.61		36.44 ± 0.51	
S8	Set 1	Before	36.12 ± 0.35 ¥	0.000	36.59 ± 0.52 ¥¥#	0.000	36.75 ± 0.49	0.086	36.38 ± 0.26	0.083
		After	37.52 ± 0.49		38.12 ± 0.55 #ƒ		36.75 ± 0.51		36.38 ± 0.39	
	Set 2	Before	36.38 ± 0.51 **	0.001	37.70 ± 0.41 ¥	0.000	36.72 ± 0.48	0.102	36.24 ± 0.36	0.078
		After	37.69 ± 0.61 *		38.47 ± 0.54		36.98 ± 0.48		36.54 ± 0.38	
	Set 3	Before	36.43 ± 0.33 **††	0.008	37.91 ± 0.52 **	0.001	36.79 ± 0.62	0.074	36.76 ± 0.35	0.566
		After	37.48 ± 0.66 **		38.54 ± 0.83		36.98 ± 0.73		36.48 ± 0.31	
S9	Set 1	Before	36.37 ± 0.72 §	0.000	36.92 ± 0.40 ¥	0.004	36.65 ± 0.64	0.224	36.33 ± 0.31	0.117
		After	37.12 ± 0.49		37.97 ± 0.52		36.94 ± 0.32		36.45 ± 0.39	
	Set 2	Before	36.47 ± 0.42 **#	0.047	37.78 ± 0.48 ¥	0.004	36.63 ± 0.51	0.089	36.56 ± 0.44	0.285
		After	37.24 ± 0.89		38.16 ± 0.84		36.55 ± 0.92		36.58 ± 0.41	
	Set 3	Before	36.48 ± 0.55 **¥	0.002	37.74 ± 0.56	0.000	36.88 ± 0.43	0.234	36.52 ± 0.41	0.156
		After	37.42 ± 0.54 *		38.38 ± 0.39		36.78 ± 0.38		36.69 ± 0.39	

* Changes from the same parameter of sets 1 (*p* < 0.05); ** Differences in regard to the same parameter of set 1. † Variations in connection with the same parameter of set 2 (*p* < 0.05). †† Alterations in relation to the same parameter of set 2 (*p* < 0.01); ¥ Differences with reference to the same parameter of the S1 (*p* < 0.05); ¥¥ Contrasts with respect to the same parameter of the S1 (*p* < 0.01); # Changes with the same parameter of the S4 (*p* < 0.05); ƒ Differences with the same parameter of the S5 (*p* < 0.05); § Divergences with the equivalent parameter of the S8 (*p* < 0.05). S1 = Session 1; S4 = Session 4; S5 = Session 5; S8 = Session 8; S9 = Session 9.

**Table 3:** HR of EG and CG in S1, S4, S5, S8, and S9.

**EG**							
			**S1**	**S4**	**S5**	**S8**	**S9**
SET 1		HR_1_	88.33 ± 19.67	97.79 ± 16.99 *	88.58 ± 16.84 †	90.69 ± 13.41 †	84.2 ± 14.59 †
		HR_5_	98.71 ± 20.69	107.65 ± 20.72 *	93.84 ± 19.35	97.47 ± 13.14	86.76 ± 14.48 *†
		HR_10_	107.33 ± 26.45	123.68 ± 18.25 *	104.53 ± 22.85 †	99.71 ± 19.19 †	96.48 ± 18.11 †
HR_R1_			72.86 ± 17.97	89.43 ± 24.41 *	80.86 ± 19.20 †	83.14 ± 15.25	73.86 ± 12.77 †
SET 2		HR_1_	90.74 ± 22.11	107.44 ± 22.91 *ƒ	100.8 ± 23.11 ƒ	96.43 ± 19.57 †	87.74 ± 15.61 †
		HR_5_	106.86 ± 14.17	116.12 ± 21.43	101.27 ± 17.56 †	100.39 ± 16.67 †ƒ	93.81 ± 11.34 †
		HR_10_	123.68 ± 17.19	127.31 ± 18.97	114.25 ± 24.33 †ƒ	116.80 ± 24.15	106.49 ± 19.08 *†ƒ
HR_R2_			87.29 ± 21.01 ƒ	89.71 ± 16.19	88.14 ± 13.86	87.43 ± 14.46	79.57 ± 23.19
SET 3		HR_1_	93.09 ± 25.55	108.98 ± 21.37	103.8 ± 23.24 ƒ¥	99.12 ± 13.7 †ƒ	98.54 ± 24.00
		HR_5_	104.91 ± 14.27	119.16 ± 19.68 *	104.27 ± 17.14 †	102.65 ± 15.55 †ƒ	94.87 ± 16.87 †
		HR_10_	123.31 ± 14.17	133.54 ± 21.34	113.3 ± 18.4 †	117.43 ± 20.89 †ƒ	108.67 ± 23.09 †ƒ
HR_R3_			88.29 ± 15.56 ƒ	101.00 ± 14.00 *	90.14 ± 16.62	86.43 ± 13.14 †	81.00 ± 23.90 †
**CG**							
			**S1**	**S4**	**S5**	**S8**	**S9**
SET 1	HR_1_		69.43 ± 10.91	69.43 ± 9.71	70.00 ± 10.33	69.86 ± 9.19	70.29 ± 9.23
	HR_5_		68.86 ± 10.06	69.43 ± 8.94	69.86 ± 9.28	69.00 ± 10.54	70.34 ± 10.29
	HR_10_		69.00 ± 9.80	70.6 ± 10.45	69.71 ± 9.71	69.00 ± 8.98	70.14 ± 9.86
HR_R1_			69.29 ± 10.19	69.43 ± 10.15	69.71 ± 8.62	70.14 ± 9.49	70.86 ± 9.89
SET 2	HR_1_		69.14 ± 9.62	70.75 ± 10.24	70.00 ± 10.21	69.29 ± 9.66	70.29 ± 9.71
	HR_5_		69.14 ± 9.79	70.71 ± 10.01	69.29 ± 10.48	68.86 ± 9.30	70.00 ± 9.07
	HR_10_		69.86 ± 8.67	71.00 ± 8.83	69.29 ± 10.21	69.29 ± 9.64	69.43 ± 9.22
HR_R2_			69.14 ± 9.17	69.71 ± 8.88	71.5 ± 7.31	69.43 ± 9.52	69.71 ± 9.27
SET 3	HR_1_		70.00 ± 11.1	71.17 ± 10.68	69.57 ± 9.73	69.57 ± 9.54	69.29 ± 9.74
	HR_5_		69.00 ± 9.47	70.00 ± 8.16	69.29 ± 9.79	70.14 ± 8.88	69.14 ± 9.30
	HR_10_		71.40 ± 10.19	70.00 ± 7.98	69.57 ± 9.29	69.71 ± 9.43	69.43 ± 10.11
HR_R3_			69.57 ± 10.05	69.71 ± 8.90	69.14 ± 9.82	70.29 ± 9.11	69.86 ± 9.41

* Differences with respect to the same parameter of S1 (*p* < 0.05). † Differences in comparison to S4 (*p* < 0.05). ƒ Intra-session changes with respect to the same parameter of set 1 (*p* < 0.05). ¥ Intra-session differences in comparison with the same value of set 2 (*p* < 0.05). S1 = Session 1; S4 = Session 4; S5 = Session 5; S8 = Session 8; S9 = Session 9; HR_1_ = HR in minute 1; HR_5_ = HR in minute 5; HR_10_ = HR in minute 10. HR_R1_ = HR in recovery 1, HR_R2_ = HR in recovery 2; HR_R3_ = HR in recovery 3.
